# Enhanced Functionality of Anti‐GPC3 CAR‐T Cells Against Hepatocellular Carcinoma Through Locoregional Administration

**DOI:** 10.1111/liv.70450

**Published:** 2025-11-21

**Authors:** Jue Wang, Jiale Qiu, Kin Ching Tsang, Zezhuo Su, Chenzi Zhang, Jun Tang, Yaofeng Wang, Chenqing Zhang, Chi‐Kong Li, Guangjin Pan, Bo Feng

**Affiliations:** ^1^ School of Biomedical Sciences, Faculty of Medicine; CUHK‐GIBH CAS Joint Research Laboratory on Stem Cell and Regenerative Medicine The Chinese University of Hong Kong Hong Kong SAR China; ^2^ Department of Orthopaedics and Traumatology, School of Clinical Medicine Li Ka Shing Faculty of Medicine, The University of Hong Kong Hong Kong SAR China; ^3^ Centre for Regenerative Medicine and Health, Hong Kong Institute of Science & Innovation Chinese Academy of Sciences Hong Kong SAR China; ^4^ Department of Paediatrics Hong Kong Children's Hospital, The Chinese University of Hong Kong Hong Kong SAR China; ^5^ Guangzhou Institute of Biomedicine and Health, Chinese Academy of Sciences Guangzhou China

**Keywords:** CAR‐T therapy, GPC3, hepatocellular carcinoma (HCC), locoregional therapy

## Abstract

**Background & Aims:**

The prognosis for patients with hepatocellular carcinoma (HCC) remains suboptimal, despite the rapid advancement of anti‐cancer immunotherapy. Chimeric antigen receptor (CAR) T cell therapy targeting glypican‐3 (GPC3) has been developed for HCC; however, clinical trials have demonstrated heterogeneous responses among patients and limited CAR‐T cell infiltration. Locoregional administration has emerged as a promising strategy for CAR‐T therapy against solid tumours, yet its potential for HCC treatment has not been thoroughly explored.

**Methods:**

In this study, we constructed anti‐GPC3 CAR‐T cells and examined their therapeutic efficacy through locoregional and systemic administration using multiple HCC xenograft mouse models.

**Results:**

Comparison of CAR‐T cell injections via portal vein and tail vein in mice with orthotopic HepG2 tumours demonstrated significantly enhanced tumour growth inhibition with locoregional CAR‐T therapy. Consistently, tumour infiltration of CAR‐T cells was significantly enhanced by portal vein injection and correlated with increased cytotoxicity, enhanced chemotaxis and reduced exhaustion of the tumour‐infiltrating CAR‐T cells compared to the tail vein injection group. Treatment with escalating CAR‐T cell dosages resulted in further improved functionality of CAR‐T cells and treatment efficacy, alongside improved liver function. Furthermore, portal vein injection exhibited superior tumour inhibition compared to tail vein injection in a metastatic model concurrently bearing orthotopic and extrahepatic tumour lesions.

**Conclusion:**

Collectively, our study demonstrates that locoregional CAR‐T therapy through the portal vein is associated with increased CAR‐T cell infiltration and improved therapeutic efficacy, offering promise for the treatment of both early‐ and late‐stage patients.

**Summary**

- Using multiple HCC xenograft mouse models, our study demonstrates that locoregional CAR‐T therapy through the portal vein is associated with increased CAR‐T cell infiltration, enhanced functionality and improved therapeutic efficacy.
- This offers promise for the treatment of both early‐ and late‐stage HCC patients using the clinically viable hepatic artery infusion approach, which is equivalent to the portal vein injection in mouse models.

## 1 Introduction

Hepatocellular carcinoma (HCC), the most prevalent form of liver cancer, is a highly aggressive and often fatal disease. Despite advances in novel treatment options, including targeted therapies and immunotherapies [1], the overall prognosis for patients with advanced HCC remains poor, underscoring the urgent need for innovative and effective therapeutic approaches [2]. In recent years, chimeric antigen receptor (CAR) T cell therapy has emerged as a promising immunotherapy strategy for haematologic malignancies [3], and growing efforts have explored its potential in treating HCC. Several therapeutic targets for CAR‐T cell therapy against HCC have been identified, among which glypican‐3 (GPC3) has been the most extensively studied due to its selectively high expression in HCC [4]. GPC3 is one of the heparan sulfate glycoproteins associated with fetal development but remains largely undetectable in adult human tissues. Notably, GPC3 is found to be highly expressed in tumour tissue samples from approximately 70% of HCC patients [5].

In hepatoma, GPC3 is detected on the surface and anchored to the cell membrane via a glycophosphatidylinositol linkage, making GPC3 a favourable target for CAR‐T cell therapy [6]. Additionally, GPC3 contributes to HCC stemness and proliferation by activating the Wnt/CTNNB signalling pathway [7]. Preclinical studies investigating anti‐GPC3 CAR‐T cell therapy in HCC have shown encouraging results, demonstrating the feasibility, safety and potential efficacy of this approach [8, 9]. However, phase I clinical trials using traditional systemic delivery of GPC3 CAR‐T cells reported a relatively low response rate among patients, with a partial response rate of < 20% [10]. The limited effectiveness of the systemically delivered anti‐GPC3 CAR‐T cells in these clinical trials prompted us to explore the potential of exploiting the liver‐specific vessel system for locoregional CAR‐T cell delivery for HCC treatment.

The liver has a dual blood supply through the hepatic artery and portal vein [11]. Preclinical studies in mouse models commonly adopt portal vein injections for locoregional drug delivery into the liver, mainly due to its technical accessibility [12, 13]. However, clinically, portal vein injection is rarely used due to its invasive nature and the associated risk. Instead, hepatic artery infusion (HAI) is a well‐established procedure in clinical settings and is broadly applied for treating liver tumours [14, 15]. The clinical HAI involves continuous or intermittent infusion of chemotherapeutic drugs directly into the hepatic artery using a pump or catheter system [16], which avoids systemic circulation and reduces associated toxicity, allowing for high concentrations of chemotherapeutic agents to be delivered directly into the tumour [16]. HAI‐mediated chemotherapy has been widely applied in clinical HCC treatment and studies on the prognosis of HCC patients without extrahepatic metastasis have reported that HAI chemotherapy contributed to significantly longer overall survival compared to systemic intravenous infusion [17]. Recent CAR‐T clinical trials for HCC treatment yielded unsatisfactory outcomes, which however, only tested the traditional intravenous infusion for CAR‐T cell delivery [9]. The potential of locoregional CAR‐T cell therapy for HCC treatment has not been well investigated, either via HAI‐based methods in clinical settings, or through portal vein delivery in preclinical mouse models.

In the current study, orthotopic mouse HCC xenografts were established and subsequent portal vein injection of anti‐GPC3 CAR‐T cells was employed to provide an alternative approach for CAR‐T cell infusion in clinical settings. Traditional intravenous injection of CAR‐T cells was performed via tail vein injection. Using these animal models, we compared the treatment efficacy between locoregional portal vein and systemic intravenous CAR‐T cell deliveries. Furthermore, the underlying mechanisms of locoregional CAR‐T cell delivery were investigated by analysing the functionality of the tumour‐infiltrating CAR‐T cells.

## 2 Results

### 2.1 Intratumoural Administration Enhanced Antitumour Efficacy of Anti‐GPC3 CAR‐T Cells Against Subcutaneous Xenografts

**FIGURE 1:**
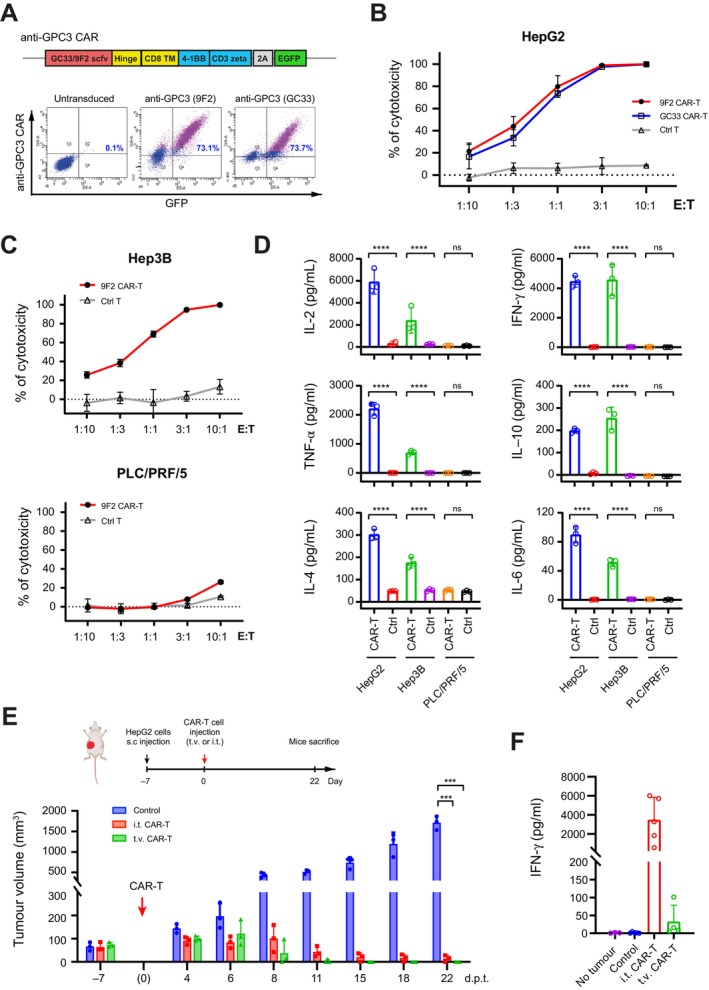
Engineering and validation of anti‐GPC3 CAR‐T cells. (A) Generation of anti‐human GPC3 CAR‐T cells. Upper: Schematics for anti‐human GPC3 CAR and GFP linked by 2A peptide. The CAR regions contain GC33 or 9F2 scFvs, hinge, CD8 transmembrane domain, 4‐1BB costimulatory and CD3 zeta signalling domain. Lower: Flow cytometry plots showing the CAR‐T cell transduction efficiency indicated by GFP expression (*x*‐axis), and CAR expression detected by GPC3 protein binding (*y*‐axis), at 7 days post‐lentiviral transduction in primary human T cells. (B) In vitro cytotoxicity of the anti‐GPC3 CAR‐T cells against HepG2 cells at different E:T ratios. Luciferase signal was measured 1 day after co‐culture of the CAR‐T cells generated and HepG2 cells carrying luciferase, at the indicated E:T ratios. Control T cells were activated but untransduced T cells. (C) In vitro cytotoxicity of 9F2 CAR‐T cells against GPC3^+^ Hep3B and GPC3^−^ PLC/PRF/5 cells at different E:T ratios. Luciferase signal was measured 1 day after co‐culture of CAR‐T cells and tumour cells carrying luciferase. Untransduced T cells were activated and used as controls. (D) The levels of cytokines secreted from CAR‐T cells after co‐culture with tumour cells at E: T ratio of 10:1. Conditioned medium was collected at 24 h post co‐inculcation, and cytokines IL‐2, IFN‐γ, TNF‐α, IL‐10, IL‐4 and IL‐6 were evaluated in three biological replicates, using the Cytometric Bead Assay (BD). The data are mean ± SD. (E) In vivo cytotoxicity of the anti‐GPC3 CAR‐T cells against subcutaneous HepG2 xenografts. Upper: Schematics showing the site of subcutaneous tumour construction in NSG mice and the workflow for the CAR‐T treatment experiments. Lower: Tumour growth curve with or without CAR‐T treatment. NSG mice bearing the subcutaneous HepG2 tumours at Day 7 post‐tumour engraftment were treated with 5 × 10^6^ CAR‐T cells/mouse via either intratumoural (i.t.) or tail vein (t.v.) injection. (F) Serum IFN‐γ levels in different treatment groups at the humane endpoint. Data shown in B–D were mean ± SD (*n* = 3). ns, not significant, with *p* > 0.05. *, *p* < 0.05.

Two anti‐GPC3 single‐chain variable fragments (scFvs), GC33 and 9F2, were sourced from prior studies to generate lentiviral CAR vectors [18, 19] (Figure 1A, upper). Human T cells transduced with the lentiviruses carrying a bicistronic CAR‐GFP cassette yielded approximately 73% GFP^+^ cells, and the expression of both GC33 and 9F2 CARs was confirmed by GPC3 antigen binding (Figure 1A, lower panels). The potent anti‐cancer cytotoxicity of these CAR‐T cells was confirmed against HepG2 cells, which express a high level of GPC3 [20] (Figure 1B). Since 9F2 CAR‐T cells displayed a trend of higher cytotoxicity compared to GC33 CAR‐T cells, it was further tested against other HCC cell lines including Hep3B and PLC/PRF/5. Notably, potent anti‐cancer cytotoxicity was confirmed against Hep3B cells which were GPC3‐positive, whereas minimal cytotoxicity was observed with PLC/PRF/5 cells which were detected as GPC3‐negative (Figure 1C; Figure S1). Corroborating these observations, cytokine analysis of the co‐culture media detected a significant increase of IFN‐γ, TNF‐α, IL‐2, IL‐4, IL‐10 and IL‐17 in the tests with HepG2 and Hep3B cells (Figure 1D), indicating a strong activation of 9F2 CAR‐T cells upon the direct exposure to GPC3 antigen. Conversely, the incubation with PLC/PRF/5 produced minimal cytokines (Figure 1D). These results demonstrated the antigen‐dependent cytotoxicity of the 9F2 CAR‐T cells.

Subcutaneous HCC xenografts were established in 4‐week‐old male NSG mice by implanting HepG2 tumour fragments (5 mm in diameter) into the right flank region (Figure 1E, upper). At Day 7 post‐tumour implantation, the tumour size reached the therapeutic window of 50–100 mm^3^, and 5 × 10^6^ anti‐GPC3 (9F2) CAR‐T cells were administered either intravenously via the tail vein (t.v.) or intratumourally (i.t.) into the tumour xenografts (Figure 1E, red arrow). Longitudinal observations from these mice indicated significant inhibition of tumour growth in both treatment groups (Figure 1E). An observed trend of faster response (Figure 1E) and a higher level of serum interferon‐gamma (IFN‐γ) detected at the endpoint in the i.t. group compared to the t.v. group (Figure 1F) suggested the potential of locoregional administration to enhance the efficacy of CAR‐T cells in treating solid tumours.

### 2.2 Portal Vein Injection of Anti‐GPC3 CAR‐T Cells Outperformed Systemic Delivery via Tail Vein, Effectively Suppressing Both Early‐Stage and Advanced Orthotopic HepG2 Tumour Growth

To test in a more clinically relevant model, we constructed orthotopic HCC xenografts in mouse liver and examined the efficacy of locoregional delivery of anti‐GPC3 CAR‐T cells through portal vein (p.v.) injection. The systemic intravenous delivery via t.v. injection was included for comparison. The locoregional distribution achieved by portal vein delivery was verified by injecting luciferase‐expressing Jurkat cells into the NSG mice. Indeed, in vivo imaging performed 1 h post‐injection showed that the cells injected via portal vein were confined to the liver, while those injected through the tail vein exhibited a first‐pass effect in the lung due to systemic delivery [21, 22] (Figure S2A). Immunostaining for CD3^+^ 9F2 CAR‐T cells in liver and lung tissue sections collected at 24 h post‐injection further corroborated these findings (Figure S2B,C).

**FIGURE 2:**
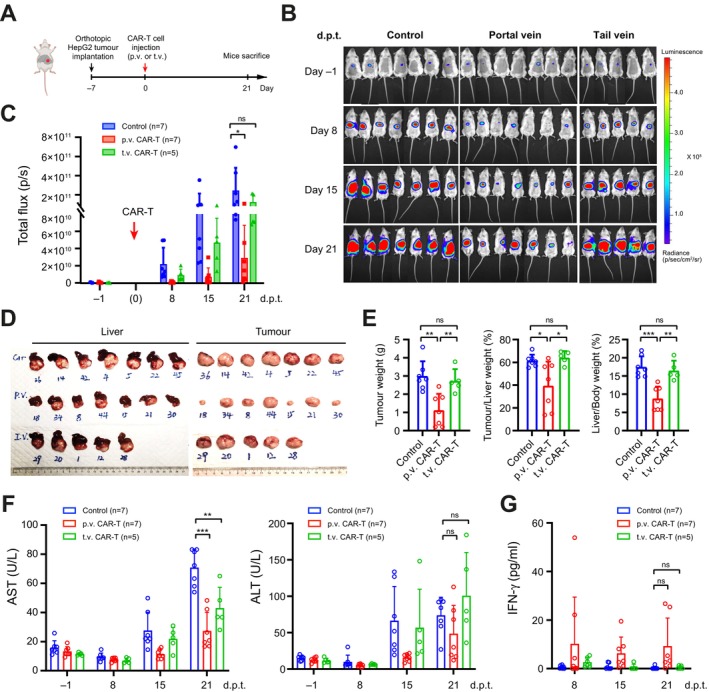
Portal vein injection of anti‐GPC3 CAR‐T cells suppressed early‐stage orthotopic HepG2 tumors. (A) Schematics showing the construction site of orthotopic tumours in NSG mice and the timeline for CAR‐T cell treatment through p.v. and t.v. injection. (B) Images of NSG mice with the bioluminescence signals produced by HepG2 tumour xenografts. 4‐week‐old NSG mice were orthotopically implanted with 1 mm^3^ HepG2 tumour fragments expressing luciferase. Tumour growths were monitored in vivo by bioluminescence imaging weekly before and after CAR‐T cell treatment. (C) The intensities of luciferase signals in B, indicating the growth of orthotopic tumours, with or without CAR‐T treatment through different administration routes. Data were collected weekly from different treatment groups and are shown at the timepoints indicated as days post‐treatment (d.p.t). (D) Images of the livers (left) and orthotopic tumours (right) at the endpoint. (E) Data of tumour weights (left), tumour/liver weight ratio (%, middle) and liver/body weight ratio (%, right) at the endpoint in different groups. (F) Levels of serum AST (left) and ALT (right) in different groups. Data were collected at different blood sampling timepoints pre‐ and post‐CAR‐T treatment. (G) Serum IFN‐γ levels in different treatment groups post CAR‐T treatment. Data shown in C, E–G represent mean ± SD (*n* ≥ 5). ns, not significant, with *p* > 0.05. *, *p* < 0.05. **, *p* < 0.01. ***, *p* < 0.001.

Next, we constructed orthotopic HCC xenografts by implanting luciferase‐expressing HepG2, Hep3B and PLC/PRF/5 tumour fragments into mouse liver. We then administered 5 × 10^6^ anti‐GPC3 CAR‐T (9F2) cells via portal vein injection on Day 7 post‐operation to mimic the treatment of small tumours representing early‐stage HCC and meanwhile, performed tail vein injection as a control for systemic delivery (Figure 2A). Tumour growth in all mice was monitored weekly using in vivo imaging and evaluated based on luciferase signal intensities. Consistent with the in vitro assay, the CAR‐T treatments against GPC3^+^ xenografts constructed using HepG2 and Hep3B cells, via either p.v. or t.v. injections, demonstrated robust anti‐tumour efficacy (Figure 2B,C; Figure S3). In contrast, the anti‐tumour effect against the GPC3^−^ xenografts composed of PLC/PRF/5 cells was negligible (Figure S4). A slower growth of Hep3B‐derived tumours was consistently observed compared to HepG2 tumours. While the CAR‐T treatments against Hep3B tumours resulted in rapid responses and nearly complete tumour eradication in both p.v. and t.v. injection groups (Figure S3), the treatment against HepG2 tumours yielded only partial tumour regression at the endpoint (Figure 2B,C). Interestingly, quantitative analysis of the tumour‐derived luciferase signals during treatment against HepG2 tumours revealed significantly lower tumour growth in p.v. groups, indicating enhanced tumour inhibition and higher CAR‐T cell efficacy compared to the t.v. injections (Figure 2B,C). A similar trend was observed in the test against Hep3B tumours, despite the rapid responses in both p.v. and t.v. groups largely masking the difference (Figure S3). Consistently, subsequent examination of the dissected HepG2 tumours and livers at the endpoint (Figure 2D) further confirmed the above findings, showing a significant reduction in tumour weight, tumour/liver weight ratio and liver/body weight ratio in the portal vein injection group compared to the systemic treatment (Figure 2E). Moreover, improved liver function and reduced liver damage were observed in the p.v. treatment correlating with enhanced tumour control, as evidenced by the lower serum aspartate aminotransferase (AST) and alanine transaminase (ALT) levels compared to the t.v. group (Figure 2F). Interestingly, similar to the intratumoural injection, portal vein injection contributed to a higher serum IFN‐γ level compared to the systemic treatment (Figure 2G), indicating enhanced activation of CAR‐T cells. Since human IFN‐γ does not cross‐react with the mouse receptor [23], the elevated human IFN‐γ levels likely exert no direct activity on mouse liver or immune cells. Instead, they reflect enhanced CAR‐T cell activation, correlating with improved therapeutic outcomes.

**FIGURE 3:**
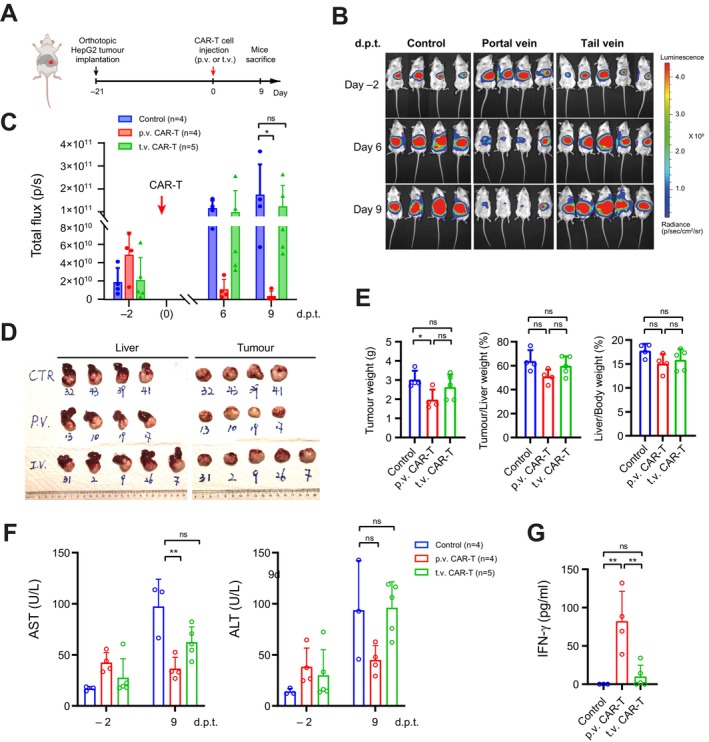
Portal vein injection of anti‐GPC3 CAR‐T cells effectively suppressed advanced orthotopic HepG2 tumors. (A) Schematics showing the construction site of orthotopic tumours in NSG mice and the timeline for CAR‐T cell treatment through p.v. and t.v. injection. (B) Images of NSG mice with the bioluminescence signals produced by HepG2 tumour xenografts. 4‐week‐old NSG mice were orthotopically implanted with 1 mm^3^ HepG2 tumour fragments expressing luciferase. Tumour growths were monitored in vivo by bioluminescence imaging weekly before and after CAR‐T cell treatment. (C) The intensities of luciferase signals in B, indicating the growth of orthotopic tumours, with or without CAR‐T treatment through different administration routes. Data were collected weekly from different treatment groups and are shown at the time points indicated as days post‐treatment (d.p.t). (D) Images of the livers (left) and orthotopic tumours (right) at the endpoint. (E) Data of tumour weights (left), tumour/liver weight ratio (%, middle), and liver/body weight ratio (%, right) in different groups at the endpoint. (F) Levels of serum AST (left) and ALT (right) in different groups. Data were collected at Day 2 pre‐ and Day 9 post‐CAR‐T treatment (d.p.t.). (G) Serum IFN‐γ levels in different treatment groups at Day 9 post CAR‐T treatment. Data shown in C, E–G were mean ± SD (*n* ≥ 5). ns, not significant, with *p* > 0.05. *, *p* < 0.05. **, *p* < 0.01. ***, *p* < 0.001.

To further examine the potential of portal vein injection of CAR‐T cells in treating advanced tumours, we postponed CAR‐T treatment after orthotopic HCC xenograft implantation so as to challenge the CAR‐T cells with a higher tumour burden. In this setting, 5 × 10^6^ anti‐GPC3 CAR‐T cells were administered on day 21 post‐implantation of tumour xenografts (Figure 3A). Notably, a significant reduction in luciferase signal was robustly observed in the portal vein injection group, indicating effective tumour burden control (Figure 3B,C). In contrast, the systemic delivery of anti‐GPC3 CAR‐T cells via tail vein injection failed to suppress tumour growth in the large orthotopic HCC xenografts (Figure 3B,C), consistent with the previous observations [19]. Endpoint examination of the dissected livers and tumours largely confirmed these findings (Figure 3D,E). Notably, in this high tumour burden test, portal vein injection was consistently associated with improved liver function and higher serum IFN‐γ levels compared to the systemic treatment group (Figure 3F,G). Collectively, these observations underscore the potential of locoregional administration as a more effective route for delivering CAR‐T cells to treat HCC and enhance overall treatment outcomes.

### 2.3 Portal Vein Injection Enhanced CAR‐T Cell Tumour Infiltration and Rendered a Higher Level of Cytotoxicity and a Lower Level of Exhaustion

**FIGURE 4:**
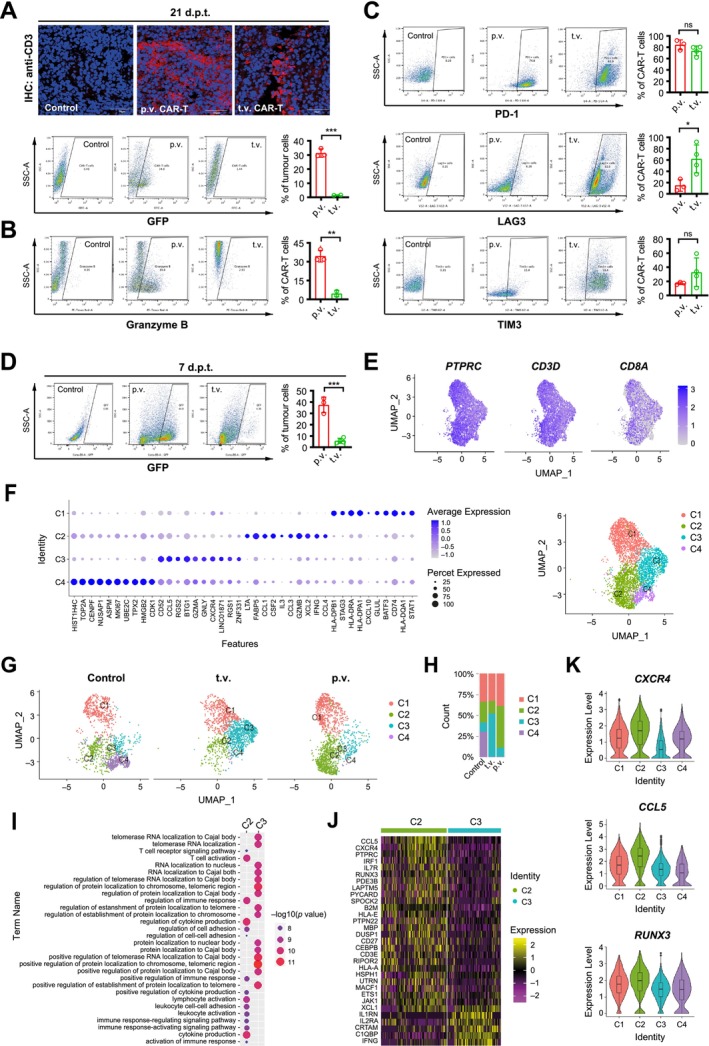
Portal vein injection enhanced CAR‐T cell tumor infiltration and rendered a higher level of cytotoxicity, a lower level of exhaustion and a higher chemotactic ability. (A) Detection of tumour‐infiltrating CAR‐T cells at the endpoint on day 21 post treatment. Upper: Immunostaining of CD3 in the orthotopic tumour tissues. Nuclei were counterstained by Hoechst 33342. Bars = 70 μm. Lower: Flow cytometry detection of GFP^+^ CAR‐T cells in the tumour cell suspensions. Shown were representative plots (left) and quantitative analysis for more than three mice in each group (*n* ≥ 3). (B, C) Flow cytometry detection for granzyme B, PD‐1, LAG3 and TIM3 among the tumour‐infiltrating CAR‐T cells. Shown were representative plots (left) and quantitative analysis for more than three mice in each group (*n* ≥ 3). (D) Flow cytometry detection of GFP^+^ CAR‐T cells in the tumour cell suspensions at Day 7 post CAR‐T treatment. (E) UMAP plots showing the expression of haematopoietic cell marker PTPRC (left), T cell marker CD3D (middle) and CD8A (right) among the tumour‐infiltrating CAR‐T cells. Colour intensity indicates expression levels. (F) Clustering analysis among the single cells. Left: Dot plot showing the average expression (colour intensity) and percentage of cells (dot size) expressing selected marker genes across the clusters. Right: UMAP plot displaying four cell clusters (C1, C2, C3, C4) identified. Each dot represents a single cell, with colours indicating different clusters. (G) UMAP plots split by treatment groups showing the distribution of cell clusters in each treatment group. (H) The bar chart indicates the relative abundance of each cluster within treatment groups. (I) Gene ontology (GO) enrichment analysis comparing C2 enriched in the portal vein injection (p.v.) group and C3 in the tail vein injection (t.v.) group. The dot/heatmap plot displaying top 10 biological processes of each group. Dot size represents gene ratio, and colour indicates −log10(*p*‐value). (J) Heatmap of differentially expressed genes (DEGs) enriched in cell adhesion (GO: 0030155) in clusters C2 and C3. Colour scale shows expression levels. (K) Violin plots displaying the expression of chemotaxis marker *CXCR4*, *CCL5* and *RUNX3* across cell clusters. The y‐axis shows normalised read counts, with box plots indicating median, quartiles, and range within 1.5 times the interquartile range. Data shown in A–D were mean ± SD (*n* ≥ 3). ns, not significant, with *p* > 0.05. *, *p* < 0.05. **, *p* < 0.01. ***, *p* < 0.001.

To investigate the mechanisms underlying the enhancement of CAR‐T cell efficacy via portal vein injection, we examined the penetration of CAR‐T cells detected in the orthotopic HepG2 tumours. Indeed, immunostaining at the endpoint (day 21 post‐CAR‐T cell treatment) revealed a significantly higher number of CD3^+^ CAR‐T cells in the tumour tissues from the portal vein injection group compared to the tail vein injection (Figure 4A, upper panels). Corroborating this observation, flow cytometry analysis of the single‐cell suspensions from collected tumours demonstrated a significantly higher proportion of tumour‐infiltrating CAR‐T cells (GFP^+^) in the tumour bulk receiving portal vein CAR‐T cell treatment compared to the tail vein injection (Figure 4A, lower panels). Additionally, granzyme B level was higher in the p.v. treatment group, indicating a more robust cytotoxic profile compared to the tail vein injection group (Figure 4B). Furthermore, we examined T cell exhaustion markers, LAG3, TIM3 and PD‐1 [24], using flow cytometry analysis. While comparable levels of PD‐1^+^ CAR‐T cells were detected in both treatment groups, significantly lower granularity was observed in the portal vein group, indicating a more naïve and pre‐exhaustion phenotype [25] (Figure 4C, upper). Consistently, much lower levels of LAG3 and TIM3 were detected among the tumour‐infiltrating CAR‐T cells collected from portal vein injection tumours compared to those from tail vein injection (Figure 4C, middle and lower panels). Collectively, these observations suggested that CAR‐T cells administered via portal vein exhibited higher tumour infiltration and cytotoxicity, while exhibiting less exhausted phenotype compared to those delivered via tail vein.

### 2.4 Portal Vein Injection Enhanced CAR‐T Cell Functionality

To further examine the features of CAR‐T cells contributing to the improved efficacy via locoregional administration, we performed single‐cell RNA sequencing (scRNA‐seq) analysis for tumour‐infiltrating CAR‐T cells. To investigate the transcriptional changes during active tumour engagement, we selected an earlier timepoint and sorted tumour‐infiltrating CAR‐T cells on Day 7 post‐treatment for scRNA‐seq. Consistently, a significantly higher penetration of CAR‐T cells was detected in the portal vein injection group compared to the tail vein injection group (Figure 4D). We observed a uniform distribution of pan‐leukocyte marker *PTPRC* (*CD45*) and T cell CD3 complex component *CD3D* at high levels, confirming the T cell identity (Figure 4E). Based on the transcriptomic profiles collected from the three sample groups, unsupervised clustering revealed four populations with distinct gene markers (Figure 4F). Notably, cluster C2 was more abundant in the portal vein injection (p.v.) group, while cluster C3 was more prevalent in the t.v. group (Figure 4G,H). Gene ontology (GO) enrichment analysis indicated that cluster C2 was associated with biological processes related to T cell activation, regulation of cytokine production and positive regulation of the immune response, suggesting a more active cytotoxic T cell function via portal vein injection (Figure 4I). Consistently, heatmap analysis revealed higher expression of genes regulating cell adhesion and chemotaxis, including *CXCR4*, *CCL5* and *RUNX3*, in the C2 cluster compared to C3 (Figure 4J,K). Collectively, these results align with the flow cytometry data at day 21 post‐treatment, indicating a more activated and functionally potent status of CAR‐T cells delivered via the portal vein [26, 27, 28]. Hence, portal vein injection significantly enhanced CAR‐T cell efficacy against orthotopic HCC xenografts by improving not only their tumour infiltration but also their functionality.

### 2.5 Higher Doses of CAR‐T Cell via Portal Vein Injection Correlated With Greater T Cell Infiltration and Less Exhaustion, Resulting in Better Efficacy

**FIGURE 5:**
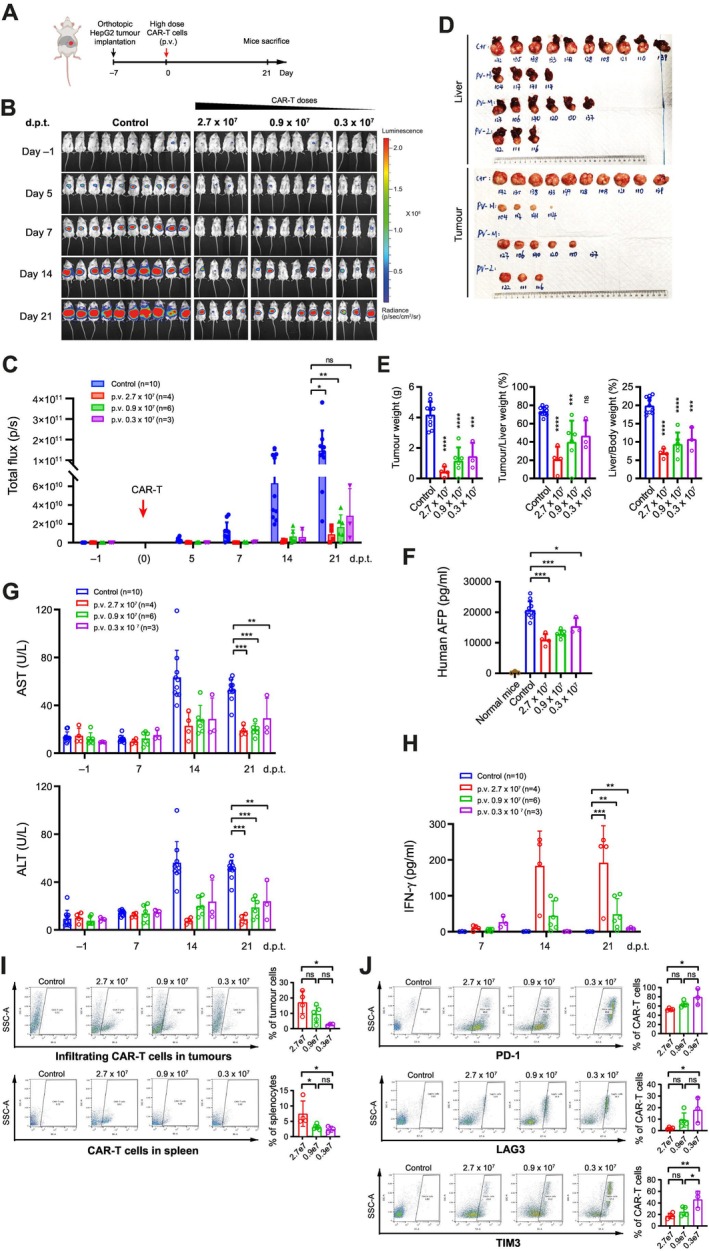
Higher doses of CAR‐T cells via portal vein injection correlated with greater T cell infiltration and less exhaustion, resulting in better efficacy. (A) Schematics for the construction site of orthotopic tumours and the timeline of CAR‐T cell treatment via p.v. injection. (B) Images of the mice in different treatment groups, with the bioluminescence signals produced by HepG2 tumour xenografts. 4‐week‐old NSG mice were orthotopically implanted with 1 mm^3^ HepG2 tumour fragments expressing luciferase. Tumour growths were monitored in vivo by bioluminescence imaging at the indicated timepoints before and after CAR‐T cell treatment. (C) The intensities of luciferase signals in B, indicating the growth of orthotopic tumours, with or without CAR‐T treatment through p.v. injection. Data from different dose groups were collected at the timepoints indicated as days post‐treatment (d.p.t). (D) Images of the livers (upper panels) and orthotopic tumours (lower panels) dissected at the endpoint. (E) Data for the tumour weights (left), tumour/liver weight ratio (%, middle), and liver/body weight ratio (%, right) in different groups at the endpoint. (F) Serum levels of human AFP in different groups at the endpoint. (G) Levels of serum AST (upper) and ALT (lower) in different groups. Data were collected at different blood sampling timepoints pre‐ and post‐CAR‐T treatment (d.p.t.). (H) Serum IFN‐γ levels at Day 7, 14 and 21 post CAR‐T treatment. (I) Flow cytometry detection of GFP^+^ CAR‐T cells in tumour cell suspensions (upper panels) and spleen (lower panels). Shown were representative plots (left) and quantitative analysis for more than three mice in each group (*n* ≥ 3). (J) Flow cytometry detection of exhaustion marker PD‐1 (upper), LAG3 (middle), and TIM3 (lower) among the tumour‐infiltrating CAR‐T cells. For each marker analysis, representative plots (left) and percentages of CAR‐T cells expressing the marker among different treatment groups (right) were shown. Data in C, E–I were shown as mean ± SD (*n* ≥ 3). ns, not significant, with *p* > 0.05. *, *p* < 0.05. **, *p* < 0.01. ***, *p* < 0.001.

Next, we examined the potential of high‐dose CAR‐T therapy via portal vein injection. A new cohort of NSG mice with orthotopic HCC xenografts at Day 7 post‐implantation was injected with escalating doses of CAR‐T cells through the portal vein (Figure 5A). The administered doses included a low dose (0.3 × 10^7^ cells/mouse), a medium dose (0.9 × 10^7^ cells/mouse) and a high dose (2.7 × 10^7^ cells/mouse). Based on weekly records from in vivo imaging, all treatment groups demonstrated robust tumour control, with better efficacy observed in the higher CAR‐T dose group (Figure 5B,C). Examination of the dissected livers and tumours at the endpoint further confirmed these observations (Figure 5D,E). Moreover, serum analysis revealed lower human AFP levels along with higher CAR‐T doses used for the portal vein injection (Figure 5F). Together, these findings indicated a dose‐dependent anti‐tumour effect among the three dose groups.

Longitudinal analysis consistently revealed lower serum AST and ALT levels in the three treatment groups compared to the controls (Figure 5G), suggesting improved liver function due to the effective control of tumour growth. Corroborating previous observations, significant elevation of serum IFN‐γ levels was detected among the mice receiving high and medium doses of CAR‐T cell treatment. Interestingly, the IFN‐γ levels in the low dose group exhibited an initial increase at 7 d.p.t. but decreased to levels comparable to the controls after 14 d.p.t., suggesting functional exhaustion (Figure 5H). Notably, the higher doses of CAR‐T cell treatment did not elevate liver damage; instead, they were associated with more stable AST and ALT levels due to better tumour control (Figure 5G). This suggests that locoregional delivery of CAR‐T cells at higher doses does not cause liver damage itself, further supporting the safety and efficacy of this approach for treating HCC.

We further dissociated the tumours and spleens from these mice collected at the endpoint. Markedly, flow cytometry analyses detected higher percentages of CAR‐T cells within the tumour bulk (Figure 5I, upper) and spleen (Figure 5I, lower) with increasing doses of CAR‐T cells injected. Conversely, the examination of exhaustion markers, including PD‐1, LAG3 and TIM3, revealed a reverse correlation, detecting lower levels of exhaustion markers in the higher dose groups (Figure 5J). Meanwhile, a greater rise in SSC was observed at lower CAR‐T doses (Figure 5J), corroborating the enhanced antigen‐dependent activation under conditions of sub‐optimal effector‐to‐tumour cell ratio [29]. Together with the dose‐dependent increase in CAR‐T cell infiltration, these findings indicate that higher doses of CAR‐T cell treatment resulted in enhanced tumour infiltration and correlated with a less exhausted phenotype. Collectively, these results suggest that the improved CAR‐T cell functionality and treatment outcomes via portal vein injection are largely attributed to the enhanced tumour infiltration due to locoregional delivery. Meanwhile, a higher input of CAR‐T cells may have the potential to yield better tumour control and improve the efficacy of CAR‐T cell therapy in clinical applications.

### 2.6 Enhanced CAR‐T Cell Functionality via Portal Vein Injection Effectively Inhibited Extrahepatic Tumour Growth

**FIGURE 6:**
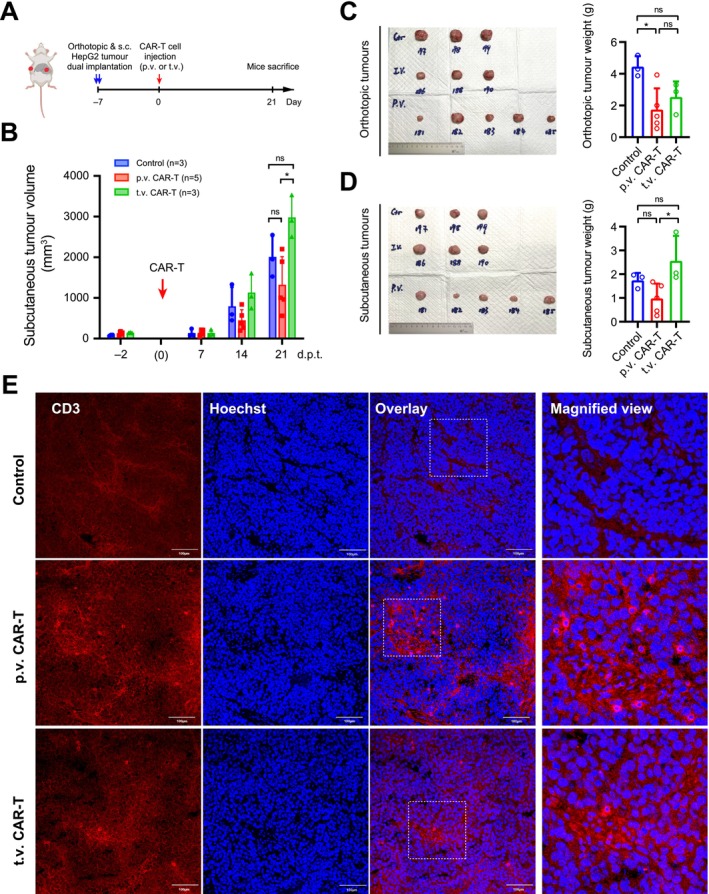
Portal vein injection inhibited metastatic tumor growth. (A) Schematics showing the construction sites of orthotopic and subcutaneous tumours in NSG mice and the timeline for CAR‐T cell treatment through p.v. or t.v. injection. (B) Growth curve of the subcutaneous tumours implanted at the flank regions. Tumour sizes were monitored weekly based on estimation. (C) Images of dissected orthotopic tumours (left) and tumour weight data (right) at the endpoint. (D) Images of dissected subcutaneous tumours (left) and tumour weight data (right) at the endpoint. (E) Immunostaining of CD3 at the endpoint in the subcutaneous tumour tissues from different groups. Nuclei were counterstained by Hoechst 33342. Bars = 100 μm. Data in A–D were shown as mean ± SD (*n* ≥ 3). ns, not significant, with *p* > 0.05. *, *p* < 0.05.

Given the improved CAR‐T cell functionality observed through locoregional administration, we further examined whether portal vein‐injected CAR‐T cells could be recruited into extrahepatic lesions, thereby exerting effective control over metastatic tumours. To address this question, we established a dual‐xenograft mouse model with tumours implanted in both the liver and subcutaneous flank regions, mimicking primary tumours and distant extrahepatic lesions, respectively (Figure 6A). Similarly, anti‐GPC3 CAR‐T cells were administered via either portal vein or tail vein for comparison. The growth of subcutaneous xenografts was monitored weekly based on the tumour sizes (Figure 6B). At the endpoint, both orthotopic and subcutaneous tumours were dissected and measured (Figure 6C,D). Consistently, portal vein injection exhibited superior tumour control over orthotopic tumours compared to tail vein injection (Figure 6C). Notably, examination of the subcutaneous tumours dissected from the flank regions also revealed better control by portal vein‐injected CAR‐T cells, while tail vein injection did not show an evident effect (Figure 6D). Immunostaining of CD3 in the subcutaneous tumour tissues detected a significantly higher number of tumour‐infiltrating CAR‐T cells in the portal vein injection group than in the tail vein injection group (Figure 6E). Collectively, our results demonstrate that CAR‐T cells locoregionally injected via the portal vein exhibit enhanced tumour‐infiltrating abilities and yield better tumour control in subcutaneous xenografts compared to systemic administration via the tail vein.

## 3 Discussion

In this study, we demonstrate that locoregional administration of anti‐GPC3 CAR‐T cells via portal vein injection significantly enhances their efficacy in treating orthotopic HCC xenografts in mouse models, compared to the traditional systemic delivery via tail vein injection. Flow cytometry and single‐cell RNA‐sequencing analysis of the infiltrating CAR‐T cells further revealed that locoregional administration not only improves CAR‐T cell infiltration into the tumour microenvironment but also augments their functionality by enhancing cytotoxicity and chemotaxis while reducing exhaustion. Tests with escalating doses of anti‐GPC3 CAR‐T cells showed a dose‐dependent increase in the treatment efficacy against orthotopic HCC tumours, with no signs of increased toxicity. Further examination in dual‐xenograft models indicated that locoregional administration of anti‐GPC3 CAR‐T cells exhibited significantly enhanced efficacy compared to systemic delivery, leading to substantial inhibition of tumour growth in both orthotopic and extrahepatic HCC xenografts. Collectively, our results provided proof‐of‐concept evidence from preclinical mouse models, supporting that locoregional administration of CAR‐T cells—achievable in clinical settings through established HAI procedures—could be a promising strategy for CAR‐T cell therapy against HCC with the potential to enhance efficacy in treating both intrahepatic and extrahepatic tumour lesions in patients.

To date, CAR‐T cell therapy for solid tumour treatment has achieved limited clinical efficacy [30]. Among the various challenges recognised [31], the inadequate infiltration of CAR‐T cells into solid tumour tissues is the most direct and detrimental cause of their ineffectiveness. To overcome this obstacle, locoregional CAR‐T cell delivery has been explored, aiming to bypass the physical barrier of solid tumours. Indeed, Katz et al. found that regional CAR‐T cell infusion was superior to systemic delivery in mouse models with peritoneal carcinomatosis [32]. Priceman et al. also reported robust efficacy through intraventricular delivery of anti‐HER2 CAR‐T cells for the treatment of multifocal brain metastases and leptomeningeal disease [33]. More recently, direct image‐guided intratumoural injection of the CAR‐T cells was tested to treat patients with HCC in a phase I clinical trial [34], though these results are not yet available. Despite these promising results, further research is necessary to identify and confirm suitable routes for locoregional CAR‐T cell delivery to treat specific solid tumours. For HCC treatment, concerns have been raised regarding the use of intratumoural injection in clinical settings, as it is impractical for treating a large number of small tumours and may be potentially ineffective. Moreover, the risk of needle tract seeding during intratumoural injection poses an additional complication [35]. These concerns prompted us to explore other routes for locoregional CAR‐T cell delivery for HCC.

Given the broad evidence supporting the safety, efficacy and technical feasibility of HAI chemotherapy for repeated infusion in clinics [36], we propose that HAI could serve as a safe route for locoregionally delivering CAR‐T cells to treat liver HCC in clinical settings, minimising the risk of needle‐tract metastasis. Directly testing HAI‐based locoregional CAR‐T therapy in mouse models is highly challenging. The mouse hepatic artery has a diameter typically < 0.3 mm, making murine HAI surgery extremely difficult and rarely reported. Instead, the diameter of the portal vein in adult mice is around 1.2 mm, which is sufficiently large for direct open surgical injection [37]. Direct portal vein injections in mouse models have demonstrated high success rates and are broadly used to investigate the efficacy of locoregional delivery‐based therapy [12, 13, 38]. These include various preclinical investigations to examine the efficacy of small molecules [39, 40] and large molecules [13] against HCC and other liver cancers. Therefore, in this study, we chose portal vein injections for locoregional administration of anti‐GPC3 CAR‐T cells into the mouse liver, for the purpose of comparing it with systemic delivery via tail vein injection. Our results demonstrated the superior efficacy of locoregional CAR‐T cell administration via the portal vein against the orthotopic HCC xenografts in mice, which can serve as preclinical proof‐of‐concept evidence, supporting further investigation of HAI‐based locoregional CAR‐T therapy in clinics to treat patients with HCC.

Preclinical HCC CAR‐T studies often utilized subcutaneous tumour models to demonstrate a significant growth inhibitory effect, ranging from 50% inhibition to complete eradication of the tumours [8, 19]. We observed similar results in our HepG2 subcutaneous tumour model, where anti‐GPC3 CAR‐T cells effectively eradicated the tumours via either intratumoural or tail vein injection routes (Figure 1E). However, referencing a few available reports from phase I clinical trials using anti‐GPC3 CAR‐T cells, CAR‐T cell therapy for HCC patients has not demonstrated much effectiveness in terms of response rates among subjects [41]. This discrepancy suggests that the commonly used subcutaneous tumour models do not adequately represent the real clinical challenges. Therefore, we adopted the orthotopic liver tumour model, which serves as a better representation of the liver microenvironment [42]. Indeed, the use of portal vein injection showed better tumour inhibition, while systemic tail vein injection of the CAR‐T cells did not curb orthotopic tumour growth (Figure 2B–E). Our data not only support orthotopic tumours as superior models to simulate clinical challenges but also demonstrate that locoregional CAR‐T cell administration could be a better approach for treating liver tumours. Moreover, in previous studies using subcutaneous models, anti‐GPC3 CAR‐T cells controlled small tumour growth but failed to inhibit tumours with a higher burden [19]. In our results, portal vein injection significantly controlled larger tumour growth at week 3 post‐implantation (Figure 3B–E), suggesting that locoregional CAR‐T cell therapy could be potentially effective in patients with higher tumour burden. Furthermore, the reduced levels of serum AST and ALT in the portal vein injection group (Figures 2F and 3F) indicated that no direct liver damage was introduced by locoregional therapy.

IFN‐γ is known to be produced by infiltrating CAR‐T cells upon antigen stimulation and has been regarded as a key factor indicating the therapeutic efficacy in clinics [43, 44]. In our study, higher levels of serum IFN‐γ were robustly detected in the p.v. CAR‐T treatment group, correlating with better CAR‐T cell infiltration into tumours (Figure 4A), compared to systemic CAR‐T cell delivery via tail vein (Figures 2G, 3G and 5H). These observations were corroborated by previous studies [45], supporting that the augmented tumour infiltration by p.v. injections contributes to higher IFN‐γ production in p.v. CAR‐T groups. Meanwhile, the immunocompromised conditions in NSG mice further supported the persistence of CAR‐T cells, resulting in prolonged IFN‐γ elevation, even after the tumours were largely eradicated [46]. In line with these observations, our experiments with escalating doses of anti‐GPC3 CAR‐T cells delivered through portal vein injection revealed a dose‐dependent increase of serum IFN‐γ levels and a consistent correlation with the enhanced tumour infiltration of CAR‐T cells at higher CAR‐T cell doses (Figure 5I,J).

Additionally, our observations also substantiate that the distinct administration routes that confine CAR‐T cells to specific circulation processes before infiltrating into the orthotopic tumours may have affected the CAR‐T cell status. Markedly, a much higher level of serum IFN‐γ was detected in the intratumoural (i.t.) CAR‐T injection group (Figure 1F), which was followed by the p.v. CAR‐T group. In contrast, t.v. CAR‐T administration demonstrated low serum interferon levels, despite its anti‐tumour effect (Figures 2G, 3G and 5H). The varied IFN‐γ levels observed among the three groups were largely correlated with the implications of administration strategies that confine CAR‐T cells to distinct circulation processes before antigen exposure. The i.t. injections could directly expose CAR‐T cells to tumour antigens, thus leading to the highest CAR‐T cell activation and IFN‐γ production. Whereas the CAR‐T cells from p.v. delivery infiltrated into the orthotopic tumours via microcirculation in the liver, and the t.v. injected CAR‐T cells passed through the systemic circulation in the whole body. The extra circulation processes might have exposed the CAR‐T cells to non‐specific stimuli, thus negatively affecting cell status and subsequent activation by tumour antigen [47]. Given that, the enhanced CAR‐T cell activation due to better functionality may also contribute to higher IFN‐γ production in p.v. CAR‐T groups than in t.v. groups.

The IFN‐γ has also been reported to play a critical role in modifying the tumour microenvironment [48], especially in myeloid activation and endogenous immunity induction [49]. However, since the human IFN‐γ does not bind to the mouse receptor [23], this mechanism is unlikely to contribute to the enhanced anti‐tumour efficiency observed in the p.v. CAR‐T group in our study. The impact of IFN‐γ on CAR‐T cell functionality is another critical issue, which however, has been extensively investigated but yields controversial observations. On one hand, high IFN‐γ levels were shown to enhance CAR‐T cell activation and its cytotoxicity [43, 48]. On the other hand, blocking IFN‐γ signalling using antibodies or knocking out the IFN‐γ receptor (IFN‐γR) in CAR‐T cells was found to enhance CAR‐T efficacy in murine models of multiple tumours [50, 51]. Hence, further research is warranted to examine the exact impact of high IFN‐γ levels on CAR‐T functionality and efficacy in p.v. groups.

In this study, single‐cell RNA sequencing analysis of the tumour infiltrating CAR‐T cells further revealed that the CAR‐T cell activation was significantly enhanced in the p.v. injection group compared to the t.v. group (Figure 4). The enhanced functionality of p.v. CAR‐T cells was indicated by the increased expression of the cytotoxicity marker granzyme B (Figure 4B) and the reduced expression of exhaustion markers LAG3 and TIM3 among the tumour‐infiltrating CAR‐T cells (Figure 4C). An enrichment in the T cell adhesion pathway was further supported by the upregulation of genes regulating T cell chemotaxis and recruitment (Figure 4J,K), including chemokine receptors *CXCR4* and *CCL5* which enable T cell migration toward tumours [52, 53]. Notably, our analysis also highlighted the upregulation of *RUNX3* (Figure 4J,K), which has been recently reported to regulate immune cell migration [28]. Wang et al. have showed that ectopic expression of *RUNX3* improved CAR‐T cell potency in solid tumour treatment [54] and this finding has been adopted in a phase I clinical trial for HCC patients [55]. The enhanced CAR‐T activation and better functionality may be directly attributed to the higher level of tumour‐infiltrating CAR‐T cells (Figure 4A), as supported by the positive impact of high CAR‐T dose (Figure 5). Additionally, bypassing systemic circulation may avoid exposing CAR‐T cells to non‐specific stimuli that bind to other immune receptors, thus preserving the CAR‐T cell in a less ‘exhausted’ status. Nevertheless, the enhanced functionality and chemotaxis profile of CAR‐T cells via p.v. injection may further contribute to the improved control over distal tumours that mimic the extrahepatic lesions potentially resulting from tumour metastasis (Figure 6). Hence, the findings of this study can potentially benefit a significant number of patients diagnosed at advanced stages with distant metastases [56].

An inherent limitation of this study was the use of immunodeficient mouse models, which were necessary for engrafting human tumour cells but did not capture crosstalk between tumour and host immunity, thus unsuitable for evaluating the implications of high IFN‐γ. Moreover, using HCC cell lines as xenograft tumours could not recapitulate the intrinsic complexity of human HCC and the associated pathological conditions, including liver cirrhosis and portal hypertension. Although our study did not consider the complications of portal hypertension, this limitation poses minimal concern because clinical evidence has established HAI as a low‐risk procedure that can be safely performed in cirrhotic patients under standard care [57, 58]. Future research employing humanised PDX models or immunocompetent mouse models has the potential to better mimic clinical situations and to address safety concerns. Additionally, further studies are warranted to investigate the underlying mechanisms behind the enhanced T cell migration following locoregional injection, as well as to examine the potential of combining targeted therapies or immune checkpoint inhibitors with locoregional CAR‐T cell therapy to further enhance the treatment efficacy against HCC.

In this study, we provided robust preclinical evidence that locoregional CAR‐T cell therapy utilising the liver vessel system is superior to traditional systemic intravenous delivery for controlling tumour growth in HCC xenografts, including early‐stage and advanced orthotopic tumours, as well as extrahepatic lesions that mimic metastatic tumours. Furthermore, tumour‐infiltrating CAR‐T cells in the locoregional therapy group exhibited higher activation, enhanced chemotaxis and a lower exhaustion phenotype. The results of this study could offer valuable insights for clinical trials and healthcare providers considering HAI‐mediated locoregional CAR‐T cell therapy for HCC patients.

## 4 Methods and Materials

### 4.1 Cell Lines and Cell Culture

The human HCC cell lines HepG2 (HB‐8065), Hep3B (HB‐8064), PLC/PRF/5 (CRL‐8024) and the human embryonic kidney cell line HEK293T (CRL‐3216) were purchased from the American Type Culture Collection (ATCC, Manassas, Virginia, USA). Human Peripheral Blood Pan‐T Cells (70024) were purchased from STEMCELL Technologies (Vancouver, British Columbia, Canada). The HepG2, Hep3B, PLC/PRF/5 and HEK293T cell lines were cultured in DMEM supplemented with 10% FBS and 1% penicillin/streptomycin, maintained at 37°C with 5% CO_2_. Human T cells were activated using Dynabeads human T activator coated with antibodies against CD3 and CD28 (11131D, ThermoFisher Scientific, Waltham, MA, USA) and cultured in RPMI‐1640 medium, supplemented with 10% FBS, 1% penicillin/streptomycin and 200 IU/mL interleukin‐2 (IL‐2). Two days post‐activation, the human T cells were transduced with a lentivirus encoding the CAR targeting GPC3 for 24 h, followed by expansion over a period of 14 days. The CAR‐T cells were then evaluated for transduction efficiency and binding affinity using flow cytometry.

### 4.2 Lentiviral Packaging of GC33 and 9F2 CARs

GC33CAR and 9F2CAR were packaged into lentivirus using HEK293T cells through polyethylenimine (PEI) transfection. HEK293T cells were seeded at a density of 3.8 × 10^6^ cells per 10 cm tissue culture dish in DMEM medium with 10% FBS and incubated at 37°C with 5% CO2 for approximately 20 h. The transfection mixture included psPAX2 (1.3 pmol), pMD2.G (0.72 pmol) and the GPC3 CAR transfer plasmid (1.64 pmol), which were combined with Opti‐MEM to a final volume of 500 μL. Separately, PEI was diluted in Opti‐MEM to achieve a DNA:PEI mass ratio of 1:3. These two solutions were gently mixed dropwise and incubated at room temperature for 12–15 min, which was then added to the HEK293T cells along with fresh medium. Viral supernatants were collected at 72 h post‐transfection, filtered (0.45 μm) and concentrated by ultracentrifugation at 25000 rpm for 2 h at 4°C. The resulting viral pellets were resuspended in PBS and stored at −80°C.

### 4.3 Establishment of Subcutaneous and Orthotopic HCC Xenograft Models

Four‐week‐old male Nod‐Scid gamma (NSG) mice were used in compliance with the guidelines approved by the Animal Experimentation Ethics Committee of The Chinese University of Hong Kong. To establish the parental subcutaneous tumour model, 5 × 10^6^ HepG2 cells were suspended in 50% Matrigel and injected subcutaneously into each mouse. Subsequently, the developed tumours were excised and cut into pieces with a diameter of approximately 3–5 mm and subcutaneously implanted into the right flank region of recipient mice. One week later, 5 × 10^6^ CAR‐T cells were administered either via tail vein injection or directly into the tumour. For the orthotopic liver tumour model, 1 mm tumour fragments were surgically implanted into the left liver lobe of recipient mice. One week post‐surgery, the mice received an injection of 5 × 10^6^ CAR‐T cells either via the tail vein or the portal vein. The mice were monitored closely for tumour growth and overall health, with regular assessments to evaluate the efficacy of the CAR‐T cell therapy in both subcutaneous and orthotopic models.

### 4.4 Mouse Portal Vein Injection

Mice were anaesthetised using Ketamine/Xylazine (100 and 10 mg/kg body weight respectively) via intraperitoneal injection and placed in the supine position. A midline incision was made in the abdomen to expose the portal vein near the liver. 5 × 10^6^ CAR‐T cells were injected into the portal vein through a 31‐gauge needle. The abdominal incision was then closed with sutures. The mice were monitored closely post operation until they recovered from anaesthesia and analgesics were given to manage pain.

### 4.5 Single‐Cell RNA‐Sequencing Analysis

Freshly dissociated tumour samples were subjected to sorting using the BD FACSAria Fusion Flow Cytometer to collect the GFP^+^ CAR‐T cells, which were then processed for single‐cell RNA sequencing. Single‐cell encapsulation and cDNA library construction were carried out using the Chromium Single Cell 3′ Reagent Kits v2 (PN‐120237, 10× Genomics, Pleasanton, California, USA) and the Chromium Single Cell A Chip Kit (PN‐120236, 10× Genomics, Pleasanton, California, USA). Cells were loaded following standard protocols to capture between 5000 and 10 000 cells per chip position for each sample. The libraries were sequenced on an Illumina NovaSeq 6000 platform, producing 150 nt paired‐end reads with 100 GB of raw data per sample.

The reads were processed using the Cell Ranger 6.1.1 pipeline and aligned to the human reference genome (GRCh38) using the STAR algorithm [59]. Gene‐barcode matrices for each sample were created by counting unique molecular identifiers (UMIs) and filtering out barcodes not associated with cells. This process resulted in gene‐cell matrices containing barcoded cells and gene expression counts. These matrices were then imported into the Seurat (v5.0.1) R toolkit for further quality control and downstream analysis [60, 61]. All functions were run with default settings, except where specified otherwise. Before integrating samples, each sample was individually inspected, and cells of low quality were filtered out (< 200 genes per cell, < 3 cells per gene, > 6000 nFeature_RNA and > 10% mitochondrial gene percentage). The fast mutual nearest neighbours (MNNs) algorithm implemented in SeuratWrappers was employed to remove batch effects and integrate samples from different groups [62]. Gene markers of individual cell clusters were filtered by min.pc < 0.25, logfc. threshold < 0.25, pct.1 > pct.2 and *p*_val_adj < 0.05. Enrichment analysis was performed using the gprofiler2 R toolkit. Functional terms with term size > 1000 were filtered out [63].

### 4.6 Statistical Analysis

The results are presented as means ± SD of at least three independent experiments. Fluorescent‐imaging analysis and animal studies were conducted in a blinded and randomised manner. The experiments shown are representative of at least three independent experiments. Microsoft Excel was used for calculations and GraphPad Prism was used to plot graphs. The sample sizes and *p* values are indicated in the figure graphs or the figure legends. Two‐tailed unpaired Student's *t*‐test was used to compare between two groups and One‐way Analysis of Variance (ANOVA) followed by Tukey's multiple comparison test was used to analyse the statistical significance for more than two groups. Differences with *p* values < 0.05 were considered statistically significant.

## Author Contributions

Jue Wang performed most of the experiments and data analysis. Jiale Qiu, Jun Tang, Kin Ching Tsang, Chenzi Zhang and Chenqing Zhang performed some of the experiments. Zezhuo Su and Yaofeng Wang performed bioinformatics analysis. Jue Wang and Bo Feng conceived the project, designed experiments, interpreted results and wrote the manuscript. Jue Wang, Bo Feng, Yaofeng Wang, Chi‐Kong Li and Guangjin Pan revised the manuscript. All authors have contributed to and approved the final paper.

## Conflicts of Interest

The authors declare no conflicts of interest.

## Supporting information


**Data S1:** liv70450‐sup‐0001‐supinfo.pdf.

## Data Availability

All data generated or analysed during this study are included in this manuscript and its Supporting Information files. The single‐cell RNA‐seq data generated in this study have been deposited in the NCBI Gene Expression Omnibus database under accession code PRJNA1148800 (https://submit.ncbi.nlm.nih.gov/subs/sra/SUB14666847/overview).
