# Supplementary material for: Enhanced Functionality of Anti‐GPC3 CAR‐T Cells Against Hepatocellular Carcinoma Through Locoregional Administration
Source: Liver Int. 2025 Nov 21;45(12):e70450. doi: 10.1111/liv.70450 (PMC12635792; doi:10.1111/liv.70450)
Supplement: Supplementary file 1 — Data S1: liv70450‐sup‐0001‐supinfo.pdf. [file LIV-45-0-s001.pdf]

Supplementary information

# Enhanced Functionality of Anti-GPC3 CAR-T Cells Against Hepatocellular Carcinoma Through Locoregional Administration

Jue WANG, Jiale QIU, Kin Ching TSANG, Zezhuo SU, Chenzi ZHANG, Jun TANG, Yaofeng WANG, Chenqing ZHANG, Chi-Kong LI, Guangjin PAN, Bo FENG

## Supplementary Methods

### Cytotoxicity assay

The cytotoxicity of anti-GPC3 CAR-T cells was assessed *in vitro* using a luciferase-based assay (E1910, Promega, Madison, WI, USA) following the manufacturer's protocol. HepG2-Luc, Hep3B-Luc, or PLC/PRF/5-Luc cells, which stably express luciferase, were co-cultured with anti-GPC3 CAR-T cells at various E:T ratios in a 96-well plate and incubated at 37°C with 5% CO2 for 24 hours. Following incubation, the cells were washed with ice-cold PBS to remove non-adherent cells and debris, followed by lysis using passive lysis buffer (PLB) for 15 minutes at room temperature. The lysates were then transferred to a fresh 96-well plate for luciferase activity measurement. First, the lysates were mixed with Luciferase Assay Reagent II (LAR II) to measure firefly luciferase activity, which indicates cell viability in xenograft tumours constructed using HepG2-Luc, Hep3B-Luc, or PLC/PRF/5-Luc post-CAR-T cell treatment. Subsequently, Stop & Glo Reagent was added to quench the firefly signal and activate Renilla luciferase, which serves as an internal control. Luminescence from both luciferases was measured using a luminometer. The relative luminescence units (RLUs) from firefly luciferase were normalized to those from Renilla luciferase to account for variations in cell number or assay conditions, with the normalized data used to quantify CAR-T cell cytotoxicity.

### Cytokine release assay

The cytokine levels were quantified using the BD™ Cytometric Bead Array (CBA) Human Th1/Th2 Cytokine Kit II (551809, BD Biosciences, NJ, USA) in accordance with the manufacturer's instructions. This kit enables the quantitative measurement of six cytokines: Interleukin-2 (IL-2), Interleukin-4 (IL-4), Interleukin-6 (IL-6), Interleukin-10 (IL-10), Tumour Necrosis Factor (TNF), and Interferon-γ (IFN-γ) in a single sample. The culture media and mouse serum samples were appropriately diluted and incubated with a mixture of six distinct capture beads, each conjugated with specific antibodies against one of the target cytokines. These beads are characterised by unique APC fluorescence intensities. Concurrently, a phycoerythrin (PE)-conjugated detection reagent, composed of PE-labelled antibodies specific to the cytokines, was added to the samples. Following incubation, the samples were analysed via flow cytometry to detect cytokine levels, with the PE fluorescence intensities indicating cytokine concentration. Data analyses were performed using FlowJo™ software.

### Tumour harvest and dissociation

To examine the tumour-infiltrating CAR-T cells, tumour tissues were processed using the Tumour Dissociation Kit, human (130-095-929, Miltenyi Biotec, Rhineland, Germany) following the manufacturer's instructions. In summary, the tumour tissues were finely minced into small pieces of approximately 2 - 4 mm and digested enzymatically for 30 min using the enzyme mix of Enzyme H, Enzyme R, and Enzyme A in DMEM. Mechanical dissociation was performed using the gentleMACS™ Dissociator, followed by incubation at 37°C under continuous rotation using the MACSmix™ Tube Rotator. The gentleMACS™ program was selected due to the texture of the tumour tissue. The resulting tumour cell suspension was filtered through a 70-μm MACS SmartStrainer and collected by centrifugation at 300 × g for 7 minutes. Red Blood cells were lysed using Red Blood Cell Lysis Solution (10×) prior to subsequent analysis.

**Flow cytometry**

Cells dissociated from tumour tissues were pelleted by centrifugation at 400 × g for 5 minutes, which were then resuspended and stained with fluorescence-conjugated antibodies (1:50 dilution). After 30 minutes of staining at room temperature, cells were washed and resuspended in a flow buffer (554656, BD Pharmingen, NJ, USA) for cytometry analysis using the BD LSRII Fortessa Flow Analyser.

**Tumour tissue processing and immunofluorescence staining**

The freshly excised tumour and liver tissues were immediately snap frozen in Tissue-Tek™ O.C.T. compound (4583, Sakura Finetek, CA, USA) to preserve cellular structure and molecular integrity. The embedded tissues were sectioned at a thickness of 8 µm using a cryostat and fixed in ice-cold 4% paraformaldehyde for 15 minutes. Following fixation, the sections were washed three times with PBS containing 0.1% Triton X-100 to permeabilise cell membranes and were blocked with 10% normal goat serum (NGS) and 0.3 M glycine for 1 hour at room temperature. Primary antibodies, diluted in antibody dilution buffer (1% NGS in PBS), were applied to the sections, which were then incubated overnight at 4°C in a humidified chamber. The sections were washed with PBS and incubated with secondary antibodies conjugated to fluorescent dyes for 1 hour at room temperature in the dark to preserve fluorescence. After washing, the sections were counterstained with Hoechst for 5 minutes. Finally, the samples were mounted using VECTASHIELD Antifade Mounting Medium (H-1000-10, Vector Laboratories, CA, USA) for fluorescent microscopy.

***In vivo* luciferase imaging**

The development of tumours was monitored weekly using the IVIS Spectrum in vivo imaging system for four weeks. Mice were weighted, anaesthetised using Ketamine/Xylazine (100 mg/kg and 10 mg/kg body weight respectively), and intraperitoneally injected with D-luciferin (GoldBio, #LUCK-100) at 0.15 mg/g body weight, followed by immediate bioluminescence imaging. The bioluminescent signals of the tumour areas were then analysed to assess tumour growth. Radiance values, indicative of tumour-produced activity, were automatically calculated by the imaging software. The scale bar's maximum and minimum values for each image were manually adjusted to be consistent to ensure comparability.

**Mouse serum AST and ALT assays**

For AST detection, 0.1 mL of mouse serum was added to a test tube, followed by 0.5 mL of pre-warmed (37°C) AST substrate solution and incubated at 37°C for 30 minutes. After incubation, 0.5 mL of DNPH solution was added to stop the reaction, incubated at room temperature for 20 minutes. Subsequently, 5 mL of 0.4 mol/L NaOH solution was added and incubated at room temperature for 5 minutes. The absorbance was then measured at 505 nm using a spectrophotometer. The procedure for ALT detection was identical to that for AST, with the use of ALT-specific substrate solution. For both AST and ALT assays, the OD values were determined against a sodium pyruvate standard curve to calculate enzyme activity (U/L).

**Human serum alpha-fetoprotein (AFP) assay**

The method for detecting human AFP in mouse serum using the Human AFP SimpleStep ELISA® Kit (ab193765, Abcam, Cambridge, UK) involves equilibrating all kit reagents to room temperature and preparing mouse serum samples by centrifuging at 2,000 x g for 10 minutes, followed by appropriate dilution. An 80 ng/mL AFP standard stock solution was prepared and serially diluted to generate a standard curve. 50 µL of standards or diluted mouse serum samples were added to the wells, followed by 50 µL of Antibody Cocktail. The plate was incubated at room temperature for 1 hour on a plate shaker, then washed three times with 350 µL 1X Wash Buffer. 100 µL of TMB Development Solution was added and incubated for 10 minutes, followed by 100 µL of Stop Solution. The absorbance was measured at 450 nm and AFP concentration were derived from the standard curve.

## Supplementary Figures

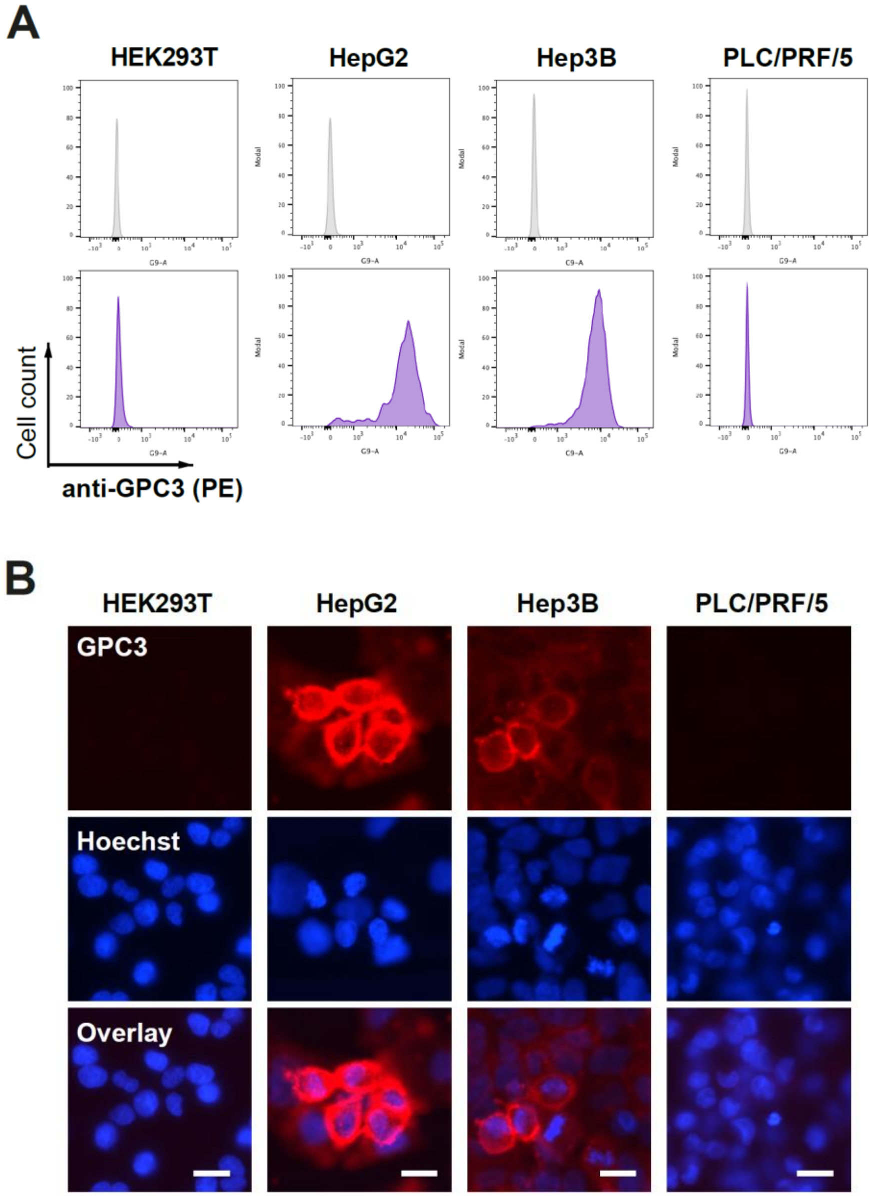


**Supplementary Figure 1. GPC3 protein expression levels in different human HCC cell lines.** (A) Flowcytometry analysis of human HCC cell lines, HepG2, Hep3B and PCL/PRF/5. Non-HCC human cell line HEK293T was used as normal control. (B) Fluorescence images from immunostaining of HepG2, Hep3B and PCL/PRF/5 cells. Primary antibody used was specific to human GPC3 (Abcam), and secondary antibody was PE-conjugated (Abcam). Nuclei were counterstained by Hoechst 33342. Bars = 20 μm.

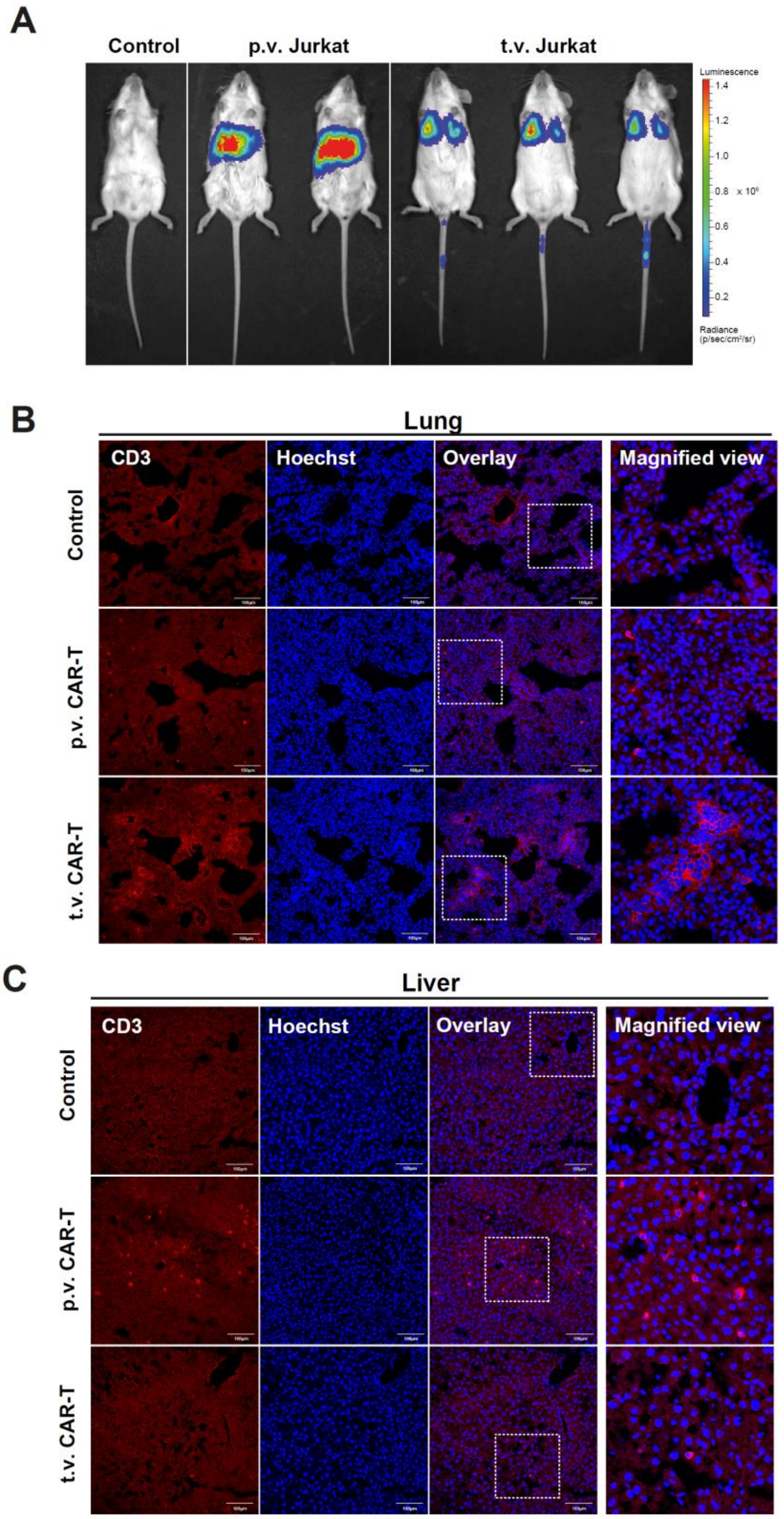


**Supplementary Figure 2. Distribution of anti-GPC3 CAR-T cells via portal vein and tail vein injections.** (**A**) Bioluminescence images of the NSG mice after receiving luciferase-expressing Jurkat cells via portal vein and tail vein injection. 4-week-old NSG mice were injected with 5 x $10^6$ cells through tail vein or portal vein. Shown were the *in vivo* imaging results at one hour after injection. (**B,C**) Immunostaining for CD3 in mouse lung and liver tissues after receiving CAR-T cell injection. 5 x $10^6$ 9F2 CAR-T cells were injected to 5-week-old tumour bearing NSG mice via tail vein or portal vein. 1 day later, mice were sacrificed for lung and liver tissue CD3 staining. Nuclei were counterstained using Hoechst 33342. Bars = 100 μm.

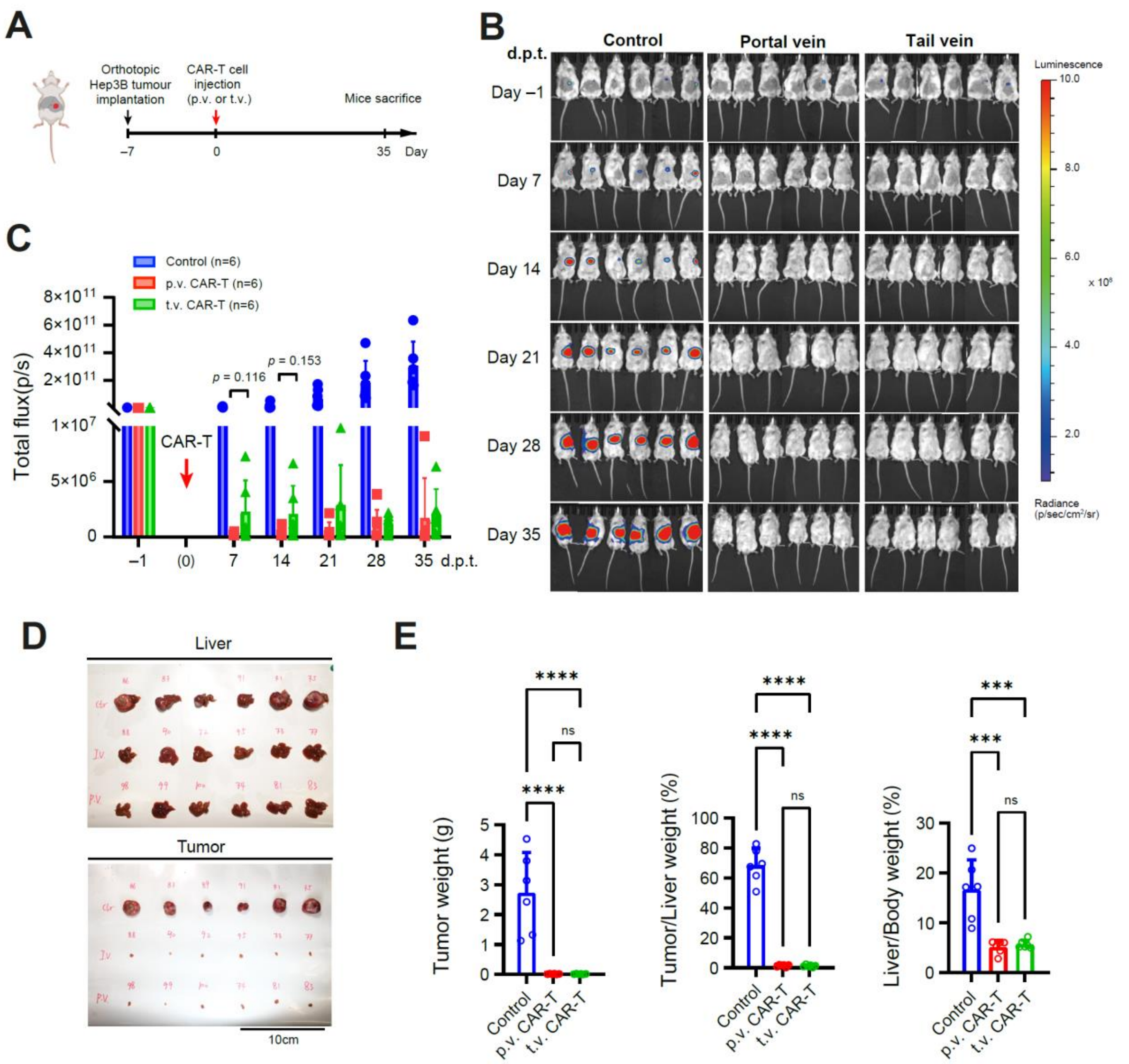


**Supplementary Figure 3. Anti-GPC3 CAR-T treatment against orthotopic Hep3B tumours via portal vein and tail vein injections.**

(**A**) Schematics showing the construction site of orthotopic tumours in NSG mice and the timeline for CAR-T cell treatment through p.v. and t.v. injections. (**B**) Images of NSG mice with the bioluminescence signals produced by Hep3B tumour xenografts. 4-week-old NSG mice were orthotopically implanted with 1 $mm^3$ Hep3B tumour fragments expressing luciferase. Tumour growths were monitored *in vivo* by bioluminescence imaging weekly before and after CAR-T cell treatment. (**C**) The intensities of luciferase signals in **B**, indicating the growth of orthotopic tumours, with or without CAR-T treatment through different administration routes. Data were collected weekly from different treatment groups and are shown at the timepoints indicated as days post-treatment (d.p.t.). (**D**) Images of the livers (upper) and orthotopic tumours (lower) at the endpoint. (**E**) Data of tumour weights (left), tumour/liver weight ratio (middle), and liver/body weight ratio (right) at the endpoint in different groups. Data in **C** and **E** represent mean ± SD (n = 6). ns, non-significant, with $p > 0.05$. *, $p < 0.05$. **, $p < 0.01$. ***, $p < 0.001$.

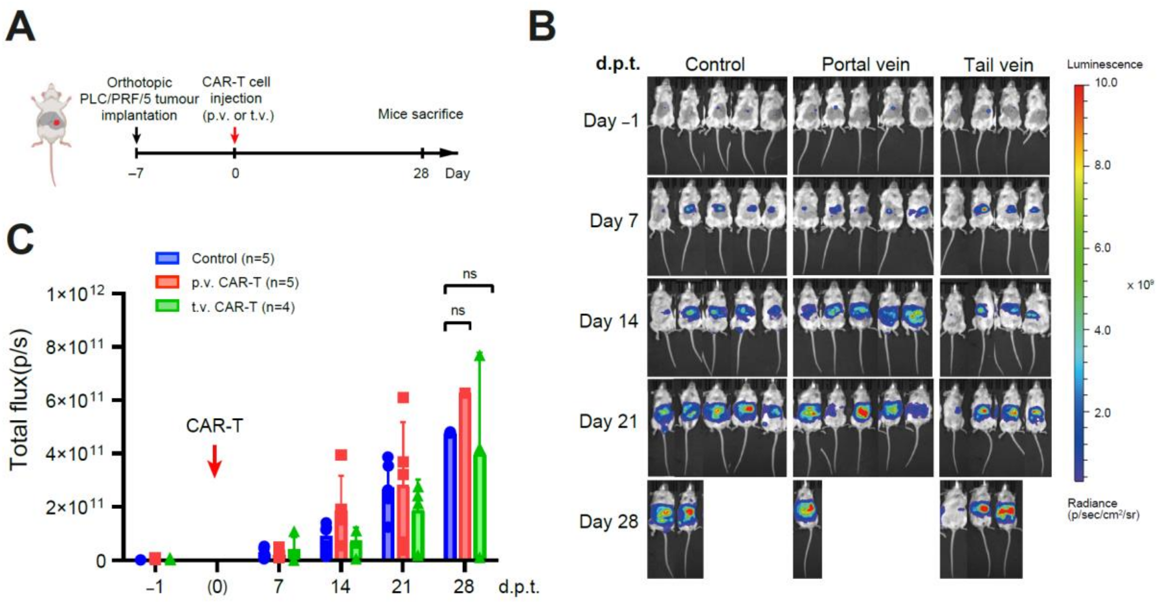


**Supplementary Figure 4. Anti-GPC3 CAR-T treatment against orthotopic PLC/PRF/5 tumours via portal vein and tail vein injections.**

(**A**) Schematics showing the construction site of orthotopic tumours in NSG mice and the timeline for CAR-T cell treatment through p.v. and t.v. injections. (**B**) Images of NSG mice with the bioluminescence signals produced by PLC/PRF/5 tumour xenografts. 4-week-old NSG mice were orthotopically implanted with 1 $mm^3$ PLC/PRF/5 tumour fragments expressing luciferase. Tumour growths were monitored *in vivo* by bioluminescence imaging weekly before and after CAR-T cell treatment. (**C**) The intensities of luciferase signals in **B**, indicating the growth of orthotopic tumours, with or without CAR-T treatment through different administration routes. Data were collected weekly from different treatment groups and are shown at the timepoints indicated as days post-treatment (d.p.t). (**D**) Images of the livers (upper) and orthotopic tumours (lower) at the endpoint. (**E**) Data of tumour weights (left), tumour/liver weight ratio (middle), and liver/body weight ratio (right) at the endpoint in different groups. Data in **C** and **E** were mean ± SD (n = 6). ns, non-significant, with $p > 0.05$. *, $p < 0.05$. **, $p < 0.01$. ***, $p < 0.001$.
